# Cumulative triglyceride-glucose-body mass index exposure and cardiovascular disease risk: findings from the Kailuan study

**DOI:** 10.3389/fcvm.2026.1670562

**Published:** 2026-07-13

**Authors:** Peng Fu, Yuxian Wang, Kuangyi Wu, Huancong Zheng, Zegui Huang, Weiqiang Wu, Zefeng Cai, Ning Wang, Hong Zheng, Haixiang Zheng, Bo Zhang, Shouling Wu, Youren Chen

**Affiliations:** 1 Department of Cardiology, Second Affiliated Hospital of Shantou University Medical College, Shantou, Guangdong, China; 2 Shantou University Medical College, Shantou, China; 3 Department of Cardiology, Sun Yat-sen Memorial Hospital of Sun Yat-sen University, Guangzhou, China; 4 Department of Cardiology, The First Affiliated Hospital of Dalian Medical University, Dalian, Liaoning, China; 5 Department of Cardiology, Kailuan General Hospital, Tangshan, China

**Keywords:** cardiovascular disease, cumulative, exposure, IR, triglyceride-glucose-body mass index

## Abstract

**Background:**

The relationship between cumulative triglyceride-glucose-body mass index (TyG-BMI) exposure and the risk of cardiovascular disease (CVD) has been unclear. This study investigated the association between cumulative TyG-BMI exposure and the risk of CVD in the Chinese population using data from the large-scale, prospective community-based Kailuan Study.

**Methods:**

The Kailuan Study included 47,577 individuals without a history of CVD or cancer who underwent health examinations in 2006, 2008, and 2010. Cumulative TyG-BMI exposure was calculated as the weighted sum of the mean TyG-BMI for each time interval (value × time). Participants were stratified into four groups based on the cumulative TyG-BMI exposure quartile. Cox proportional hazards regression models were established to calculate hazard ratios and 95% confidence intervals for evaluation of the relationship between cumulative TyG-BMI exposure and risk of CVD. The area under the receiver operating characteristic (ROC) curve was calculated to compare the predictive power of cumulative TyG-BMI, TyG, and BMI for CVD.

**Results:**

A total of 3,514 incident cardiovascular events occurred during a median follow-up of 10 years. The risk of CVD increased with increasing cumulative TyG-BMI exposure quartile. After adjusting for potential confounders, Cox regression analysis yielded respective hazard ratios (95% confidence intervals) of 1.32 (1.18–1.49), 1.33 (1.18–1.49), and 1.44 (1.29–1.62) for the Q2, Q3, and Q4 groups in comparison with the Q1 group. The subgroup analysis showed a significant interaction between cumulative TyG-BMI index and age or hypertension, but there was no interaction between sex, Diabetes mellitus and cumulative TyG-BMI index. The restricted cubic spline analysis revealed a significant non-linear relationship between cumulative TyG-BMI index and the risk of CVD. The area under the ROC curve (AUC) of cumulative TyG-BMI was 0.6047, demonstrating modestly higher discriminative performance than TyG (AUC: 0.5602) and BMI (AUC: 0.5612).

**Conclusions:**

High cumulative TyG-BMI exposure is associated with an increased risk of CVD. The TyG-BMI value may help to identify individuals at high risk of developing CVD.

## Background

Worldwide, cardiovascular disease (CVD) is the leading cause of death, accounting for 18 million deaths a year (1). For decades, public health recommendations by the American Heart Association and American College of Cardiology have focused on health promotion strategies (2, 3). However, preventative strategies are also needed to reduce the risk and burden of CVD.

Insulin resistance (IR) is known to be the mechanism underlying development of type 2 diabetes, and emerging evidence suggests it is also an independent risk factor for CVD (4). As the gold standard method for assessing insulin resistance, hyperinsulinemic euglycemic clamp is inconvenient and expensive to perform. Therefore, the triglyceride-glucose (TyG) index has been developed as a surrogate marker of IR (5). It has been shown that the TyG index has strong correlations with hyperinsulinemic euglycemic clamp test and homeostasis model assessment of insulin resistance (6, 7). A TyG index has been validated as an effective predictor of cardiovascular outcomes including acute myocardial infarction (MI), coronary artery disease, heart failure (HF), and hypertension in previous studies (8–12). Additionally, recent studies have shown that combining TyG with obesity indices [such as body mass index [BMI], waist circumference [WC], and waist-to-height ratio [WHtR]] significantly improves the accuracy of IR estimates (12). The triglyceride-glucose-body mass index (TyG-BMI) value in particular has demonstrated high consistency with the homeostasis model assessment of IR value in assessment of IR (13) and is simple and easy to perform in the clinical setting. The TyG-BMI value has also been found to be closely associated with cardiovascular outcomes in patients with non-alcoholic fatty liver disease and those with coronary artery disease (9, 14). However, most studies of the relationship between the TyG-BMI level and disease have focused on single measurements, and research on the long-term association between fluctuations in TyG-BMI and the risk of CVD is limited.

The aim of this study was to determine the association between the cumulative TyG-BMI exposure and the risk of CVD in the Chinese population using data from the Kailuan Study.

## Methods

### Study population

**Figure 1:**
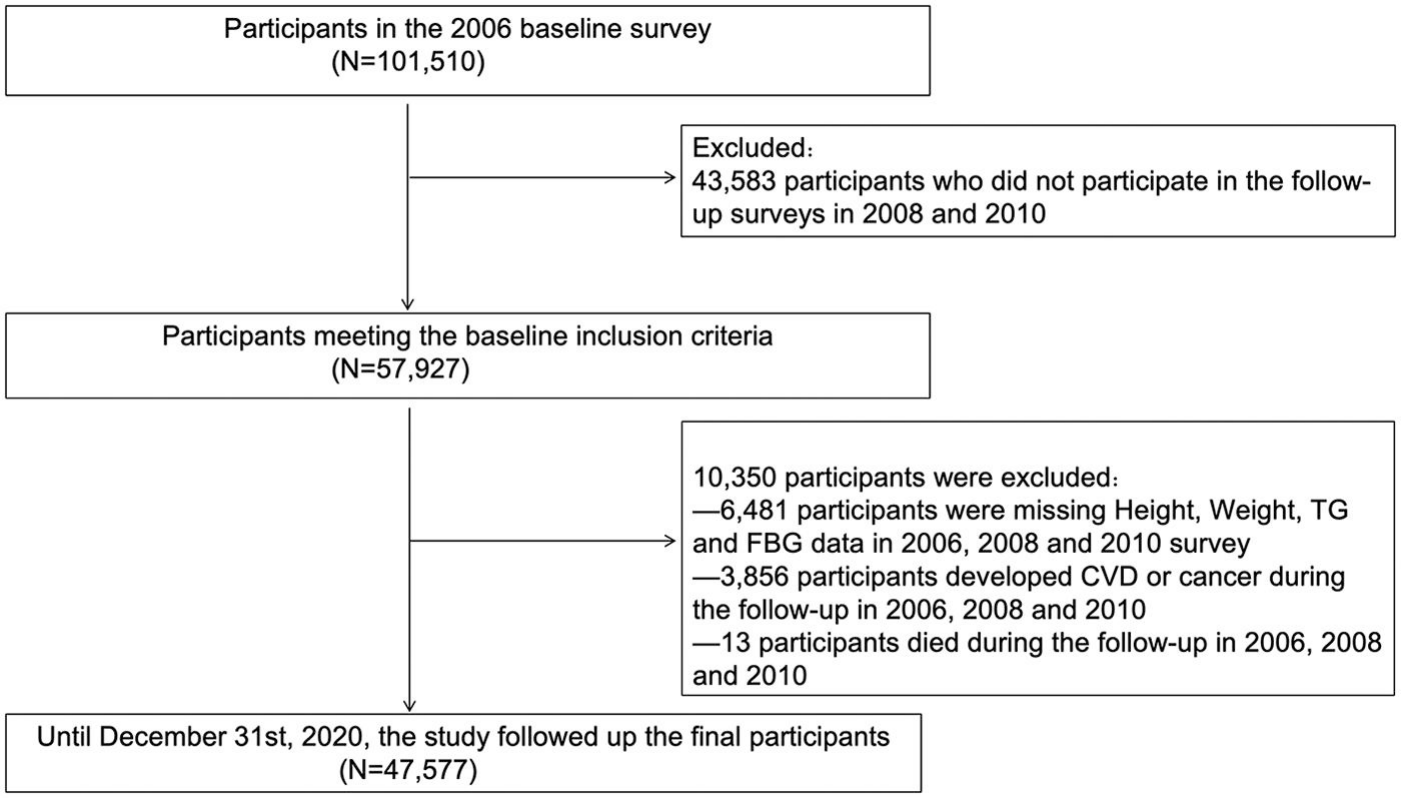
Flow chart showing the process used to select the study participants and the exclusion criteria.

The data analyzed in this study were sourced from the ongoing community-based Kailuan Study being conducted in Tangshan, China, which has a longitudinal prospective design and includes a cohort of patients recruited between June 2006 and October 2007. A total of 101,510 participants participated in baseline surveys with follow-up examinations every 2 years. Data on participants' demographic characteristics, medical history, lifestyle factors, and biomedical indicators are collected by questionnaire surveys, physical examinations, and laboratory tests. The design of the Kailuan Study and its methodology have been described in detail elsewhere (15). Eight rounds of health assessments have been completed so far. The present study included participants in the Kailuan Study who met the following criteria: three health examinations completed between 2006 and 2010; at least three complete sets of data on triglycerides (TG), fasting blood glucose (FBG), height, and weight available; and written informed consent provided. Information on CVD and cancer was obtained from the databases of hospitals belonging to the Kailuan Medical Group and participants' self-reported physician-diagnosed records. Participants with a record of cardiovascular events or tumors before or at the third examination (2010–2011) were excluded. Follow-up for incident CVD events started after the third examination. Finally, data for 47,577 participants were included in the analysis (Figure 1). This study was approved by the Kailuan General Hospital Ethics Committee and was conducted according to the Declaration of Helsinki.

### Data collection and definitions

Participants completed the survey at 11 hospitals in the Kailuan community. Standard questionnaires were used to collect data on age, sex, smoking, alcohol intake, physical activity, and medical history. Detailed information on the data collection procedures can be found elsewhere (16). For the anthropometric measurements, participants were instructed to wear light clothing and stand barefoot. Trained physicians measured weight and height under standardized conditions according to a standardized protocol, with precision to 0.1 kg and 0.1 cm, respectively. BMI was calculated as weight (kg) divided by the square of height (m^2^). Participants were categorized as non-smokers or current smokers, with current smokers defined as those who had smoked at least one cigarette per day on average in the past year. Participants were classified as non-drinkers or current drinkers, with current drinkers defined as those who had consumed ≥100 milliliters of alcohol per day on average in the past year. Active physical exercise was defined as exercise occurring at least four times per week, with each session lasting a minimum of 20 min. Hypertension was defined as systolic blood pressure (SBP) ≥ 140 mmHg and/or diastolic blood pressure (DBP) ≥ 90 mmHg, a documented history of hypertension, and/or use of antihypertensive medication (17). Diabetes mellitus was defined as a fasting blood glucose concentration ≥7.0 mmol/L, a documented history of diabetes mellitus, and/or use of Hypoglycemic drugs (18).

### Biochemical measurements and calculation of cumulative TyG-BMI exposure

Participants were instructed to fast overnight (for at least 8 h) before morning fasting venous blood samples (5 mL) were collected from the antecubital vein and placed into vacuum tubes containing EDTA. All blood samples were analyzed using a Hitachi 747 automated analyzer (Hitachi, Tokyo, Japan) and then processed in the central laboratory of Kailuan General Hospital. Laboratory tests included high-sensitivity C-reactive protein (hs-CRP), FBG, TG, high-density lipoprotein cholesterol (HDL-C), and low-density lipoprotein cholesterol (LDL-C). Creatinine levels were measured using a creatinine assay kit (BioSino BioTechnology and Science Inc., Beijing, China) (19). According to the modified Chronic Kidney Disease Epidemiology Collaboration equation, the estimated glomerular filtration rate (eGFR) was calculated using four variables (20).

The TyG index was calculated as ln [TG (mg/dL) × FBG (mg/dL)/2] (21). Next, according to the TyG × BMI formula, TyG was multiplied by BMI to obtain the TyG-BMI (composite index). Cumulative TyG-BMI exposure was calculated as follows: (TyG-BMI index_2006_ + TyG-BMI index_2008_)/2 × time 1–2 + (TyG-BMI index_2008_ + TyG-BMI index_2010_)/2 × time 2–3, where the respective TyG-BMI values in 2006, 2008, and 2010 were calculated at the first, second, and third clinic visits, and times 1–2 and 2–3 represent the specific time intervals between consecutive examinations (in years) (22).The mean values of time1–2 and time2–3 were 2.07 and 1.97 years, respectively. Follow-up for incident CVD events began after the third examination (2010–2011), following completion of the cumulative TyG-BMI exposure assessment. Participants with a history of CVD or cancer before the third examination were excluded. Participants were followed until the occurrence of CVD, death, or December 31, 2021, whichever came first.

### Definition of cardiovascular disease

CVD was defined as MI, stroke, or HF. Diagnostic information for CVD includes all participants in the Kailuan Study, which is retrieved annually from municipal social insurance institutions and discharge registries, with updates during follow-up. For all suspected cardiovascular events, an expert panel rigorously reviews annual discharge records to confirm the diagnoses. MI is diagnosed in accordance with the World Health Organization criteria for monitoring CVD trends and determinants across multiple countries. Diagnosis relies primarily on a combination of individual clinical symptoms, electrocardiographic findings, and dynamic changes in cardiac enzyme levels. Diagnostic information was retrieved from participants' discharge registries and resting electrocardiograms during each survey period (23). MI was defined using the ICD-10 code I21. Diagnoses of stroke are based on neurological signs, clinical symptoms, and neuroimaging studies, such as computed tomography and magnetic resonance imaging (24). The stroke events were identified using ICD-10 codes: ischemic stroke (I63) and hemorrhagic stroke (I60-I61). HF is diagnosed in accordance with the European Society of Cardiology standards and determined by specialist physicians based on clinical symptoms and findings on echocardiography, chest radiography, and electrocardiography (25). We used ICD-10 revision code I50.x to identify HF cases (26).

### Statistical analysis

Normally distributed continuous data are presented as the mean ± standard deviation, and non-normally distributed data as the median (interquartile range). Categorical data are shown as the frequency (percentage). When comparing the characteristics of the cumulative TyG-BMI exposure, chi-square tests were used for categorical data and one-way analysis of variance for continuous data. Non-normally distributed continuous variables are presented as the median with 25th–75th percentiles (Q1–Q3) and compared among groups using the Kruskal–Walli's test. Normality of continuous variables was evaluated using the Kolmogorov–Smirnov test. The Kaplan–Meier method was used to calculate cumulative event rates, and the log-rank test was used to compare the differences between groups. Missing data were assessed for all baseline covariates. The overall proportion of missing values was minimal, with each covariate having less than 5% missing data (Supplementary Table S1). To minimize potential bias and retain statistical power, missing values were handled using multiple imputation by chained equations (MICE). All covariates included in the multivariable models were used in the imputation models. Ten imputed datasets were generated, and results were combined using Rubin's rules to obtain pooled estimates and standard errors. Event incidence rates are expressed as events per 1,000 person-years and were calculated by dividing the number of events by the total person-years of follow-up.

After confirming the proportional hazards assumption, we performed multivariable Cox proportional hazards regression analysis to assess the independent risk association between the cumulative TyG-BMI value and CVD (MI, stroke, and HF). To account for potential confounders, we adjusted for sex and age in model 1, and then adjusted further for HDL-C, LDL-C, hs-CRP, eGFR, current smoking status (yes/no), current alcohol status (yes/no), physical activity (yes/no), education level (high school or above), hypertension (yes/no), diabetes mellitus (yes/no), use of anti-hypertensive drugs (yes/no), use of hypoglycemic drugs (yes/no), and use of lipid-lowering drugs (yes/no). These covariates were chosen by examining choice covariates in relevant literature. The variance inflation factors (VIFs) for all covariates ranged from 1.01 to 1.63, with an overall average VIF of 1.30 (Supplementary Table S2). Based on the VIF, covariate multicollinearity was not present in the final models.

A restricted cubic spline (RCS) model was employed to explore the potential non-linear association between cumulative TyG-BMI and the risk of CVD, MI, stroke, and HF.

Receiver operating characteristic (ROC) curves were constructed, and the area under the curve (AUC) was calculated to compare the predictive performance of cumulative TyG-BMI, TyG, and BMI for CVD, MI, stroke, and HF. Optimal cut-off values for each parameter were determined using the Youden Index.

Subgroup analyses were then conducted after stratifying participants by age (<60 years vs. ≥60 years), sex (male vs. female), hypertension status (yes/no), and diabetes mellitus status (yes/no). The robustness of the results was tested with multiple sensitivity analyses. Participants who experienced outcome events within the first year of follow-up were excluded to minimize the potential for reverse causality. Considering the potential influence of various medications on outcomes, three separate sensitivity analyses were then conducted by excluding participants using anti-hypertensive, hypoglycemic, and lipid-lowering drugs.

All statistical analyses were performed using *SAS* version 9.4 software (*SAS* Institute Inc, Cary, NC, USA) and *R* software, version 4.3.0 (*R* Foundation, Vienna, Austria). Two-tailed *P* < 0.05 were considered statistically significant.

## Results

### Baseline characteristics

The study cohort included 47,577 participants. Table 1 shows their baseline characteristics according to cumulative TyG-BMI exposure quartile. The mean age was 52.79 ± 11.89 years, with 23.1% of participants being female. Participants with higher cumulative TyG-BMI exposure tended to be older than those with a lower exposure. Differences in baseline characteristics were observed according to quartile, with participants in the higher quartiles having slightly elevated systolic and diasolic blood pressure, higher total cholesterol and LDL-C levels, lower HDL-C and eGFR levels, an elevated hs-CRP level, and a greater likelihood of having a history of diabetes mellitus and hypertension than those in the lowest cumulative TyG-BMI exposure quartile.

**Table 1:** Comparison of baseline characteristics according to cumulative TyG-BMI exposure quartile (*N* = 47,577).

Variables	Total	Quartiles of cumulative TyG-BMI index				*P* value
		Q1 (527.06–898.24)	Q2 (898.24–1,019.63)	Q3 (1,019.63–1,156.18)	Q4 (1,156.18–2,233.69)	
*N*	47,577	11,894	11,894	11,895	11,894	
Age, years	52.79 ± 11.89	49.19 ± 11.65	52.21 ± 11.46	54.02 ± 11.65	55.76 ± 11.80	<0.0001
Male	36,569 (76.90)	8,290 (69.70)	9,359 (78.70)	9,585 (80.60)	9,335 (78.50)	<0.0001
BMI, kg/m^2^	25.10 ± 3.37	22.02 ± 2.21	24.31 ± 2.19	25.89 ± 2.39	28.19 ± 3.13	<0.0001
Systolic blood pressure, mmHg	130.54 ± 19.01	122.39 ± 17.01	129.10 ± 17.70	132.99 ± 18.33	137.66 ± 19.54	<0.0001
Diastolic blood pressure, mmHg	84.23 ± 10.74	80.16 ± 10.05	83.66 ± 10.14	85.47 ± 10.35	87.64 ± 10.98	<0.0001
HDL-C, mmol/L	1.55 ± 0.48	1.67 ± 0.48	1.57 ± 0.52	1.51 ± 0.44	1.45 ± 0.44	<0.0001
LDL-C, mmol/L	2.60 ± 0.84	2.39 ± 0.77	2.59 ± 0.84	2.67 ± 0.82	2.77 ± 0.87	<0.0001
FBG, mmol/L	5.64 ± 1.67	5.21 ± 1.02	5.45 ± 1.47	5.69 ± 1.55	6.19 ± 2.23	<0.0001
TyG, mmol/L	10.29 ± 0.67	9.89 ± 0.53	10.19 ± 0.57	10.39 ± 0.63	10.69 ± 0.69	<0.0001
TyG-BMI_2006_	257.36 ± 44.18	215.11 ± 26.54	246.12 ± 28.12	268.09 ± 31.11	300.10 ± 38.62	<0.0001
TyG-BMI_2008_	255.95 ± 43.39	211.59 ± 23.31	242.88 ± 23.38	266.52 ± 25.99	302.82 ± 36.32	<0.0001
TyG-BMI_2010_	258.97 ± 43.01	217.93 ± 25.54	247.62 ± 26.08	269.00 ± 29.24	301.34 ± 38.77	<0.0001
Cum TyG-BMI	1,033.78 ± 192.35	806.25 ± 66.31	957.03 ± 34.59	1,080.60 ± 38.87	1,291.25 ± 121.41	<0.0001
TG, mmol/L	1.29 (0.91–1.91)	0.97 (0.70–1.32)	1.20 (0.90–1.68)	1.40 (1.01–2.08)	1.76 (1.23–2.65)	<0.0001
hs-CRP, mg/L	1.06 (0.50–2.56)	0.80 (0.39–2.00)	0.95 (0.40–2.27)	1.11 (0.50–2.60)	1.50 (0.73–3.17)	<0.0001
eGFR, mL/min	89.77 ± 18.52	93.85 ± 18.53	89.66 ± 18.53	88.10 ± 18.28	87.47 ± 18.07	<0.0001
Hypertension, *N* (%)	21,271 (44.70)	3,145 (26.4)	4,758 (40.00)	5,991 (50.40)	7,377 (62.00)	<0.0001
Diabetes mellitus, *N* (%)	4,947 (10.40)	392 (3.30)	778 (6.54)	1,347 (11.30)	2,340 (20.40)	<0.0001
Anti-Hypertensive Drugs, *N* (%)	5,024 (10.60)	540 (4.54)	939 (7.89)	1,345 (11.30)	2,200 (18.50)	<0.0001
Hypoglycemic drugs, *N* (%)	1,807 (3.80)	138 (1.16)	287 (2.41)	494 (4.15)	888 (7.47)	<0.0001
Lipid-lowering drugs, *N* (%)	406 (0.85)	43 (0.36)	91 (0.77)	100 (0.84)	172 (1.45)	<0.0001
Physical activity, *N* (%)	6,711 (14.10)	1,379 (11.60)	1,614 (13.60)	1,797 (15.10)	1,921 (16.20)	<0.0001
High school or above, *N* (%)	2,798 (15.30)	708 (15.40)	706 (15.40)	694 (15.10)	690 (15.00)	<0.0001
Current drinker, *N* (%)	6,019 (32.80)	1,464 (31.90)	1,388 (30.30)	1,494 (32.60)	1,673 (36.50)	0.0002
Current smoker, *N* (%)	18,218 (38.30)	4,595 (38.60)	4,876 (39.30)	4,674 (39.30)	4,273 (35.90)	<0.0001

The data are presented as the mean ± standard deviation, median (interquartile range), or number (percentage).

TyG, triglyceride-glucose index; Cum TyG-BMI, cumulative triglyceride-glucose-body mass index.

### Relationship between cumulative TyG-BMI exposure and risk of CVD

**Figure 2:**
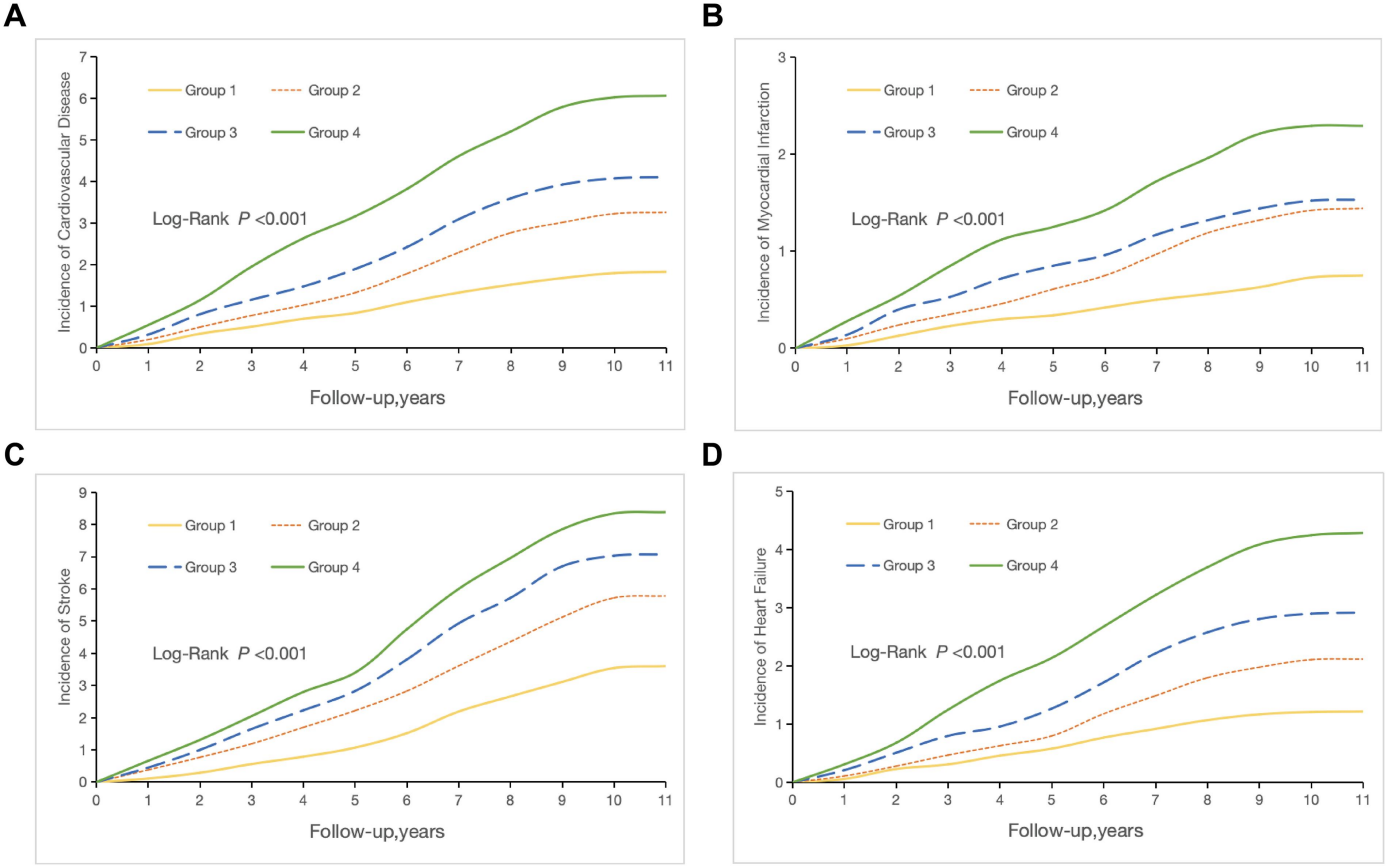
Cumulative incidence of cardiovascular disease based on cumulative TyG-BMI exposure: **(A)** cardiovascular disease, **(B)** myocardial infarction, **(C)** stroke, and **(D)** heart failure.

During a median follow-up period of 10.00 years (interquartile range: 9.61–10.32 years), 3,514 participants experienced CVD events, including 601 cases of MI, 3,031 cases of stroke, and 1,055 cases of HF. The incidence density of CVD and its subgroups are presented in Table 2. The incidence rates of CVD in the Q1, Q2, Q3, and Q4 groups were 4.15, 7.22, 8.73, and 11.61 per 1,000 person-years, respectively. Kaplan–Meier curves revealed a gradual increase in CVD incidence rates from the Q1 group to the Q4 group. The log-rank test revealed significant differences in the cumulative incidence rate for CVD and subgroup among the groups (Figure 2).

**Table 2:** Hazard ratios calculated for cardiovascular disease in the study population based on cumulative TyG-BMI exposure between 2006 and 2010.

Category	Q1	Q2	Q3	Q4	*P*-trend	Per SD increased
CVD						
Case/Total	483/11,894	817/11,894	970/11,895	1,244/11,894	–	–
IR	4.15	7.22	8.73	11.61	–	–
Model 1	1.00	1.48 (1.32, 1.66)	1.63 (1.46, 1.82)	2.01 (1.81, 2.24)	<0.001	1.27 (1.23–1.31)
Model 2	1.00	1.32 (1.19, 1.49)	1.34 (1.18, 1.41)	1.47 (1.31, 1.64)	<0.001	1.12 (1.09–1.16)
Model 3	1.00	1.32 (1.18, 1.49)	1.33 (1.18, 1.49)	1.44 (1.29, 1.62)	<0.001	1.11 (1.08–1.15)
MI						
Case/Total	75/11,894	144/11,894	153/11,895	229/11,894	–	–
IR	0.61	1.20	1.29	1.99	–	–
Model 1	1.00	1.64 (1.24, 12.17)	1.62 (1.22, 2.13)	2.34 (1.80, 3.05)	0.056	1.31 (1.21–1.41)
Model 2	1.00	1.41 (1.06, 1.86)	1.25 (0.94, 1.66)	1.56 (1.18, 2.06)	0.062	1.12 (1.03–1.23)
Model 3	1.00	1.40 (1.06, 1.86)	1.24 (0.94, 1.65)	1.54 (1.16, 2.04)	0.070	1.12 (1.03–1.22)
Stroke						
Case/Total	439/11,894	697/11,894	869/11,895	1,026/11,894	–	–
IR	3.88	6.32	7.60	8.65	–	–
Model 1	1.00	1.28 (1.12, 1.46)	1.49 (1.32, 1.70)	1.62 (1.43, 1.83)	<0.001	1.18 (1.13–1.22)
Model 2	1.00	1.22 (1.07, 1.40)	1.39 (1.22, 1.58)	1.45 (1.26, 1.65)	<0.001	1.13 (1.08–1.18)
Model 3	1.00	1.22 (1.07, 1.39)	1.38 (1.21, 1.57)	1.43 (1.25, 1.64)	<0.001	1.13 (1.08–1.18)
HF						–
Case/Total	122/11,894	212/11,894	292/11,895	429/11,894	–	–
IR	1.05	1.76	2.47	3.75	–	–
Model 1	1.00	1.44 (1.15, 1.80)	1.77 (1.43, 2.19)	2.35 (1.92, 2.87)	<0.001	1.35 (1.27–1.43)
Model 2	1.00	1.31 (1.05, 1.64)	1.50 (1.21, 1.87)	1.77 (1.43, 2.20)	<0.001	1.21 (1.14–1.29)
Model 3	1.00	1.30 (1.04, 1.63)	1.49 (1.20, 1.85)	1.72 (1.39, 2.13)	<0.001	1.19 (1.12–1.27)

Model 1: adjusted for age and sex.

Model 2: additionally adjusted for HDL-C, LDL-C, hs-CRP, eGFR, current drinker, current smoker, physical activity, education level, hypertension, and diabetes mellitus.

Model 3: adjustment of model 2 further for use of hypoglycemic drugs, anti-hypertensive and lipid-lowering drugs.

Caption: CVD, cardiovascular disease; MI, myocardial infarction; HF, heart failure; IR, Incidence rate, per 1,000 person-years.

The independent impact of cumulative TyG-BMI exposure on the risk of CVD was examined in three Cox proportional hazards regression models. In model 3, after adjusting for potential confounders, compared with the Q1 group, the respective HRs and 95% CIs in the Q2, Q3, and Q4 groups were 1.32 (1.18–1.49), 1.33 (1.18–1.49), and 1.44 (1.29–1.62). Among the CVD subtypes, the outcomes for stroke and HF followed a trend like that of overall CVD, whereas the results for MI were largely non-significant. When analyzed as a continuous variable, for each standard deviation increase in cumulative TyG-BMI, the risk of CVD increases by 11% (95% CI: 1.08–1.15), MI risk increases by 12% (95% CI: 1.03–1.22), stroke risk increases by 13% (95% CI: 1.08–1.18), and HF risk increases by 19% (95% CI: 1.12–1.27). These findings suggested that the risk of CVD was higher in participants with higher cumulative TyG-BMI exposure.

**Figure 3:**
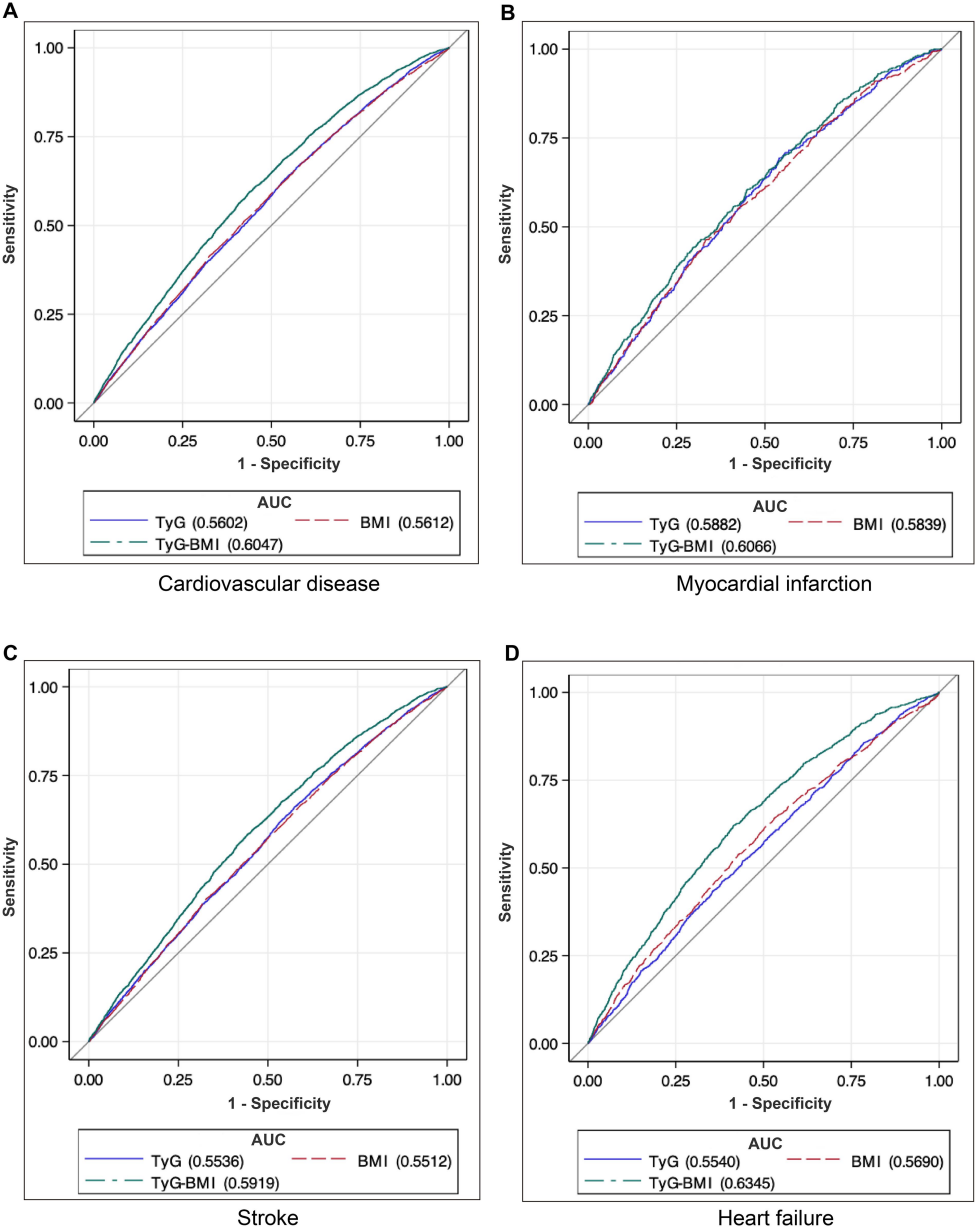
Restricted cubic spline hazard regression plot depicting the relationship between cumulative TyG-BMI and the risk of incident cardiovascular disease. Cardiovascular disease **(A)**, myocardial infarction **(B)**, stroke **(C)**, and heart failure **(D)**. TyG-BMI, triglyceride-glucose-body mass index.

The RCS analysis revealed a significant nonlinear relationship between cumulative TyG-BMI and the risks of CVD, MI, stroke, and HF. The *P*-values for the nonlinear effects indicated significant nonlinearity for all conditions except MI (*P* for non-linearity = 0.167) and HF (*P* for non-linearity = 0.100), with significant nonlinearities observed for CVD (*P* for non-linearity < 0.001) and stroke (*P* for non-linearity = 0.006). Furthermore, the risk ratio for CVD and stroke was relatively low at lower cumulative TyG-BMI levels, but showed an upward trend at higher levels (Figure 3).

### Receiver operating characteristic curve analysis

**Figure 4:**
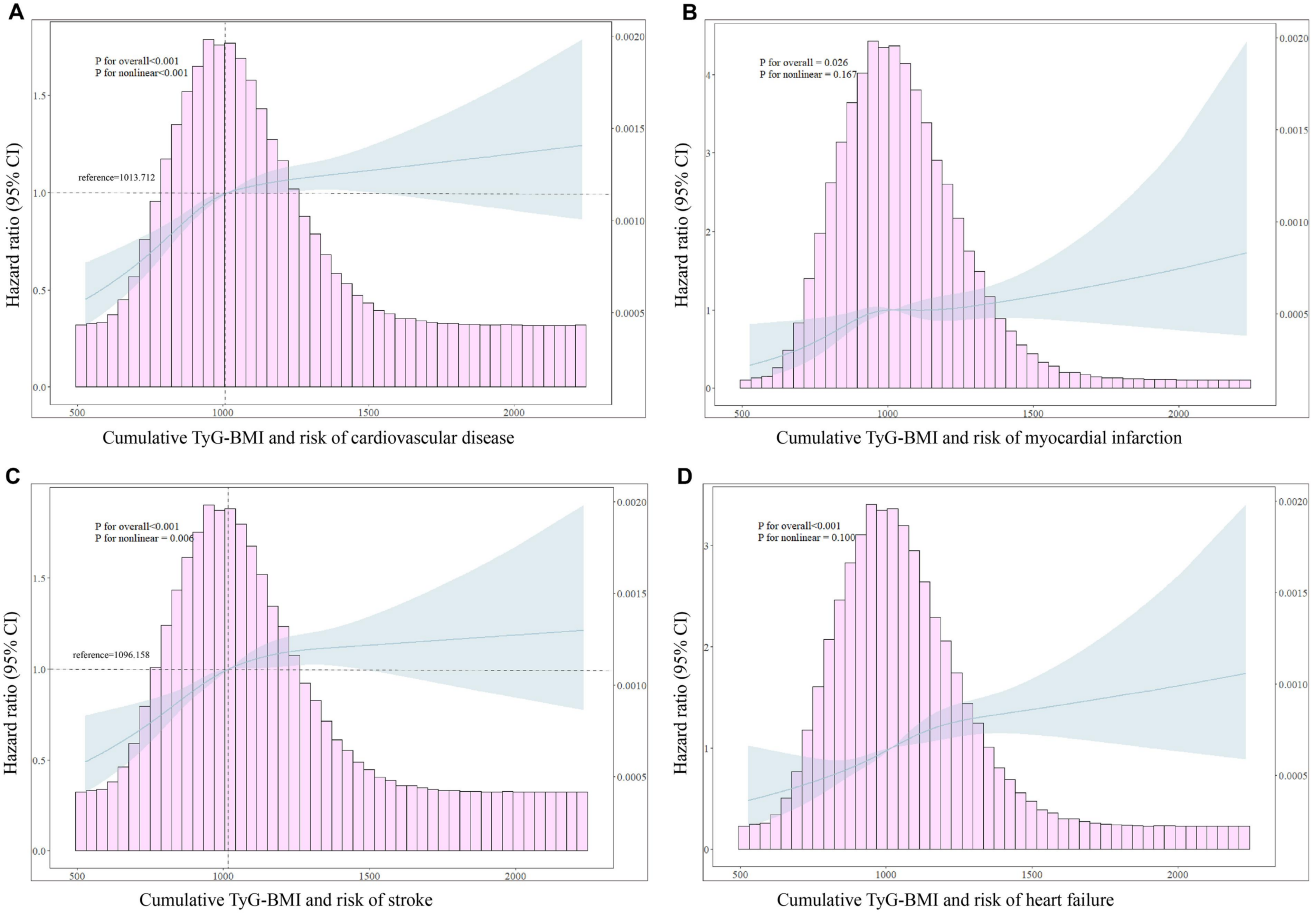
ROC curves for identifying cardiovascular disease **(A)**, myocardial infarction **(B)**, stroke **(C)**, and heart failure **(D)**, using the TyG, BMI, and the TyG-BMI. Differences in AUC among the indices were assessed using the DeLong test. AUC, Area under the curvea; TyG, triglyceride-glucose index; BMI, body mass index; TyG-BMI, cumulative triglyceride-glucose-body mass index.

According to time-dependent ROC curve analysis, cumulative TyG-BMI demonstrates good diagnostic ability in predicting the risks of CVD, MI, stroke, and HF. Compared to TyG (AUC: 0.5602-0.5882) and BMI (AUC: 0.5512-0.5839) alone (Supplementary Table S2), the AUC value of the TyG-BMI combined model is improved across all diseases, particularly in HF, where the AUC of cumulative TyG-BMI is 0.6345, significantly higher than that of TyG. These findings indicate that the combination of TyG and BMI provides superior diagnostic performance over time in predicting these diseases. The best cut-off value of cumulative for predicting CVD was 1,042.03 (Figure 4).

### Subgroup results and sensitivity analysis

The relationship between cumulative TyG-BMI exposure and CVD was stratified by age, sex, presence of hypertension, and presence of diabetes mellitus (Table 3). Cumulative TyG-BMI exposure showed a significant interaction with age and presence of hypertension but not with sex or presence of diabetes mellitus. The results of sensitivity analysis with adjustments for covariates and exclusion of participants who experienced outcome event within 1 year (Supplementary Table S3) were consistent with our main findings. Furthermore, the results were not altered after exclusion of participants using anti-hypertensive, hypoglycemic, or lipid-lowering drugs **(**Supplementary Table S4).

**Table 3:** Subgroup analysis: the hazard ratio for cardiovascular disease events in the study population based on cumulative TyG-BMI exposure between 2006 and 2010.

Subgroups	Q1	Q2	Q3	Q4	*P* for interation	Per SD increased
Age					0.0229	
Age < 60	237/8,853	427/8,851	543/8,853	674/8,853	–	–
Case/Total	1.00	1.37 (1.18, 1.61)	1.53 (1.32, 1.78)	1.60 (1.38, 1.87)	–	1.26 (1.15–1.35)
Age ≥ 60	257/3,041	358/3,042	480/3,042	538/3,042	–	–
Case/Total	1.00	1.02 (0.99, 1.18)	1.18 (1.03, 1.36)	1.19 (1.03, 1.37)	–	1.16 (1.11–1.23)
Sex					0.6453	
Male	472/9,142	694/9,142	814/9,142	1,028/9,143	–	–
Case/Total	1.00	1.21 (1.08, 1.36)	1.29 (1.15, 1.44)	1.40 (1.25, 1.56)	–	1.22 (1.18–1.36)
Female	34/2,752	110/2,752	140/2,752	222/2,752	–	–
Case/Total	1.00	1.45 (1.02, 2.08)	1.48 (1.04, 2.10)	1.78 (1.26, 2.53)	–	1.52 (1.21–1.98)
Hypertension					0.0056	
Yes	347/5,318	512/5,317	667/5,318	838/5,318	–	–
Case/Total	1.00	1.08 (0.97, 1.22)	1.16 (1.03, 1.30)	1.20 (1.07, 1.35)	–	1.13 (1.05–1.26)
No	148/6,575	225/6,577	345/6,577	412/6,577	–	–
Case/Total	1.00	1.34 (1.10, 1.63)	1.53 (1.26, 1.86)	1.75 (1.44, 2.12)	–	1.39 (1.13–1.60)
Diabetes mellitus					0.0554	
Yes	141/1,237	195/1,237	129/1,236	269/1,237	–	–
Case/Total	1.00	1.16 (0.95, 1.42)	1.08 (0.88, 1.33)	1.15 (0.93, 1.41)	–	1.07 (0.85–1.25)
No	410/10,657	739/10,659	799/10,657	832/10,657	–	–
Case/Total	1.00	1.46 (1.18, 1.79)	1.52 (1.24, 1.86)	1.87 (1.53, 2.28)	–	1.62 (1.26–1.75)

The model was adjusted for age, sex, HDL-C, LDL-C, hs-CRP, eGFR, current drinker, current smoker, physical activity, education level, hypertension, diabetes mellitus, hypoglycemic drugs, anti-hypertensive and lipid-lowering drugs.

## Discussion

To our knowledge, few prospective studies have investigated the association between cumulative TyG-BMI exposure and incident cardiovascular disease. Extending previous evidence on cumulative TyG and specific cardiovascular outcomes, our study evaluated the relationship between cumulative TyG-BMI exposure and overall CVD risk, as well as its major subtypes (MI, stroke, and HF), in a large Chinese prospective cohort. The main findings of this study are as follows: (1) higher cumulative TyG-BMI exposure is associated with an increased risk of cardiovascular events, and a significant non-linear association was observed for CVD and stroke; (2) even after adjusting for potential confounding factors, a significant association between high cumulative TyG-BMI exposure and CVD persists; (3) the ROC curve analysis showed that cumulative TyG-BMI provided higher discriminative performance than TyG or BMI alone. Similar results were observed for CVD subtypes, and the findings remained consistent in sensitivity analyses. Therefore, cumulative TyG-BMI exposure may serve as a key indicator for the prevention and management of CVD risk.

IR is a key feature of metabolic syndrome and obesity, both of which are closely linked to an increased risk of CVD (27). A Swedish cohort study (28) found that IR patients had the highest risk of cardiovascular events. The TyG index, a simple and cost-effective marker of IR (29), is associated with higher risks of MI, HF, and ischemic stroke (4, 30, 31). Obesity, which also contributes to CVD risk through conditions like HF and coronary artery disease (32), triggers chronic low-grade inflammation and plaque instability, further worsening CVD. BMI, a common measure of obesity, is a known prognostic factor for CVD, with cardiovascular mortality increasing by 5% for each unit increase in BMI (33). The TyG-BMI level, combining TG (34), FBG, and BMI, offers a more comprehensive assessment of IR and metabolic health than individual biomarkers. Recent studies highlight its strong correlation with mortality and severity of coronary artery disease (35), as well as its role as an independent risk factor for multi-vessel coronary artery disease (36). Overall, the TyG-BMI is a valuable, cost-effective tool for identifying high-risk individuals and assessing IR.

Elevated TyG-BMI levels have been associated with higher risks of atrial fibrillation, heart failure, mortality, and poor prognosis in stroke patients, according to several studies (37–39). One study also found a strong correlation between high TyG-BMI and overall mortality in stroke patients (40). A single-center study (41) involving 1,438 patients undergoing percutaneous coronary intervention and drug-eluting stent implantation showed that higher TyG-BMI levels were associated with a greater incidence of major adverse cardiovascular events. However, these studies measured TyG-BMI only once, without considering its changes over time. In contrast, our study focuses on the long-term cumulative exposure to TyG-BMI and identifies it as an independent risk factor for CVD. We found that cumulative TyG-BMI exposure more accurately predicts CVD risk, with the highest risk in participants with the highest cumulative exposure. The association between cumulative exposure to other IR-related indices and CVD risk further supports our findings (4, 42). The time-dependent ROC curve analysis also demonstrated that the cumulative TyG-BMI model demonstrated modestly higher discriminative performance than TyG or BMI alone in predicting CVD, MI, stroke, and HF, with a notable improvement in AUC, especially for HF (AUC = 0.6345). This suggests that combining TyG and BMI enhances predictive accuracy, particularly for conditions like HF, where early and accurate prediction is crucial. Our results highlight the importance of long-term monitoring of TyG-BMI levels in clinical practice to better prevent CVD.

It should be noted that the discriminative ability of cumulative TyG-BMI was modest, with all AUC values below 0.65, indicating limited utility as a standalone prediction tool. Moreover, cumulative TyG-BMI was compared only with TyG and BMI rather than established cardiovascular risk prediction models. Therefore, these findings should be interpreted as evidence of incremental predictive value beyond its individual components rather than superiority over existing clinical risk assessment tools. Future studies are needed to evaluate its additive value in established risk prediction models.

Previous studies have mainly explored the relationship between TyG indices and CVD in diabetic or non-diabetic populations. A cross-sectional study by Dang et al. found that TyG-WC, TyG-WHtR, and TyG-BMI could improve CVD prediction (43). However, conflicting evidence exists, with some studies suggesting that TyG outperforms TyG-WHtR in predicting coronary heart disease and stroke (44, 45), while others indicate that TyG-WC is more effective in diabetic and prediabetic individuals, and TyG-WHtR better identifies early CVD risk in diabetic patients (46). These discrepancies may be attributed to variations in glycemic classification. Our findings show that TyG-BMI has stronger predictive power for stroke and HF in individuals with normal glycemic status, but no significant association was found in diabetic patients. This aligns with a study in Chinese populations, which found that TyG and its derivatives have lower predictive ability in diabetic individuals (47). The lack of association in diabetics may result from pre-existing metabolic abnormalities, blood glucose control, medications, or comorbidities. Further studies are needed to clarify these findings.

It is noteworthy that the associations between cumulative TyG-BMI exposure and individual CVD subtypes were not entirely consistent. Significant associations were observed for stroke and HF, whereas the association with MI was comparatively weaker. This difference may reflect the varying contributions of insulin resistance and metabolic dysfunction to the development of specific cardiovascular outcomes. These findings highlight the importance of evaluating CVD subtypes separately and suggest that cumulative TyG-BMI may influence cardiovascular risk through both shared and outcome-specific pathways.

Our study found a stronger association between cumulative TyG-BMI exposure and CVD risk in individuals aged <60 years compared to those aged ≥60 years, highlighting age as a key factor. This may be due to the rising prevalence of obesity, dyslipidemia, and metabolic syndrome among younger adults (48). A recent study reported a 47% (49) increase in overweight and obesity rates in youth, underscoring the need for early lipid control and weight management. Additionally, a prospective cohort study showed that while obesity in middle age increases HF risk in old age, its impact on HF in later life is less pronounced (50). These findings suggest that managing lipid and metabolic abnormalities in younger populations may help prevent new-onset CVD. In conclusion, our study highlights the significant link between cumulative TyG-BMI exposure and increased CVD risk, with age as a critical factor, particularly for younger individuals. Further research is needed to confirm the impact of age on this relationship.

RCS analysis revealed a significant nonlinear relationship between cumulative TyG-BMI and the risks of CVD and stroke, whereas no significant nonlinearity was observed for MI and HF. Risk remained low at lower TyG-BMI levels but increased markedly at higher levels, highlighting the importance of monitoring elevated TyG-BMI. These findings may help clinicians identify high-risk individuals and guide personalized risk management strategies.

Although we found an association between cumulative TyG-BMI exposure and the risk of CVD, the pathological mechanisms underlying this association remain unclear. Nevertheless, several explanations have been proposed. First, research has confirmed that IR is associated with oxidative stress, endothelial dysfunction, and chronic inflammation, thereby accelerating the progression of atherosclerosis (51–53). Secondly, prediabetes and chronic hyperglycemia can promote endothelial dysfunction, inflammation, fibrosis, and cardiac remodeling by forming advanced glycation end products and increasing oxidative stress (54, 55). Several pathological processes are associated with the development and progression of cardiovascular events. Overall, the explanations we currently provide are based on the present understanding of IR-induced metabolic dysfunction and the pathophysiology of CVD. Further research is needed to elucidate the complex interactions among these factors and to delineate the underlying mechanisms in detail.

### Advantages and limitations

This study has several strengths. First, it was based on a large prospective community-based cohort with a median follow-up of 10 years and repeated measurements of TyG-BMI. By using cumulative TyG-BMI exposure, we were able to better characterize long-term metabolic burden and more precisely evaluate its association with incident CVD. Nevertheless, several limitations should be considered. First, although we adjusted for a wide range of potential confounders, residual or unmeasured confounding factors, including genetic susceptibility, cannot be completely excluded. In addition, cumulative TyG-BMI exposure was derived from three examinations conducted between 2006 and 2010. Although repeated measurements may better reflect sustained metabolic exposure than a single assessment, they may not fully capture long-term fluctuations in metabolic status. Moreover, TyG-BMI trajectories during the subsequent follow-up period were not assessed and may have influenced the observed associations. Similarly, all covariates were measured at baseline and treated as fixed variables throughout follow-up. Changes in medication use, body weight, glycemic control, and other clinical characteristics during follow-up were not captured. Second, the study population was derived from the Kailuan cohort, which predominantly consists of male Chinese workers. Although no significant interaction by sex was observed, the substantially smaller number of female participants may have limited the statistical power to detect potential sex-specific associations. In addition, our findings have not yet been externally validated in independent cohorts. Therefore, caution is warranted when generalizing these findings to female populations, other ethnic groups, occupational settings, or populations with different demographic characteristics. Finally, participants with a history of cancer were excluded to minimize potential confounding, which may have slightly limited the generalizability of the findings.

## Conclusion

This study found a close association between high cumulative TyG-BMI exposure and an increased risk of CVD. Furthermore, prolonged exposure to high TyG-BMI indices may elevate the risk of CVD, emphasizing the importance of long-term monitoring of TyG-BMI exposure in clinical practice for prevention of CVD.

## Data Availability

The original contributions presented in the study are included in the article/Supplementary Material, further inquiries can be directed to the corresponding author/s.
