# Supplementary material for: Cumulative triglyceride-glucose-body mass index exposure and cardiovascular disease risk: findings from the Kailuan study
Source: Front Cardiovasc Med. 2026 Jul 13;13:1670562. doi: 10.3389/fcvm.2026.1670562 (PMC13402370; doi:10.3389/fcvm.2026.1670562)
Supplement: Supplementary file 1 [file Datasheet1.docx]

**Supplemental Material**

**Supplemental Tables**

Table S1. Missing covariates

Table S2. Variance Inflation Factors for all covariates included in the multivariable model

Table S3. Predictive power of TyG, BMI, and TyG-BMI for cardiovascular disease

Table S4**.** Sensitivity analysis excluding outcome events occurring within the first year of follow-up (n=227)

Table S5. Sensitivity analysis excluding participants using antihypertensive, antidiabetic, and lipid-lowering medications

**Table S1. Missing covariates**

| Covariate | Number of Missing Values | Percentage of Missing Values |
| --- | --- | --- |
| LDL-C, mmol/L | 68 | (0.15%) |
| hs-CRP, mg/L | 97 | (0.20%) |
| Physical activity, N (%) | 39 | (0.08%) |
| High school or above, N (%) | 69 | (0.15%) |
| Current drinker, N (%) | 326 | (0.69%) |
| Current smoker, N (%) | 41 | (0.09%) |

Caption: LDL-C, low-density lipoprotein cholesterol; and hs-CRP, high-sensitivity C-reactive protein.

**Table S2. Variance Inflation Factors for all covariates included in the multivariable model**

| **Covariables** | **VIF** |
| --- | --- |
| Age, years | 1.63 |
| Sex | 1.32 |
| HDL-C, mmol/L | 1.12 |
| LDL-C, mmol/L | 1.05 |
| hs-CRP, mg/L | 1.01 |
| eGFR, ml/min | 1.39 |
| Current smoker, N（%） | 1.54 |
| Current drinker, N（%） | 1.52 |
| Physical activity, N（%） | 1.04 |
| High school or above, N（%） | 1.13 |
| Hypertension, N（%） | 1.33 |
| Diabetes mellitus, N（%） | 1.61 |
| Hypoglycemic drugs, N（%） | 1.02 |
| Anti-Hypertensive Drugs, N（%） | 1.25 |
| Lipid-lowering drugs, N（%） | 1.54 |

Caption: VIF, Variance Inflation Factor.

**Table S2. Predictive power of TyG, BMI, and TyG-BMI for cardiovascular disease**

|  | **AUC (95% CI)** | **Cut-off** | **Sensitivity and specificity, %** | **Youden index** | ***P* for comparison** |
| --- | --- | --- | --- | --- | --- |
| **CVD** |  |  |  |  |  |
| TyG | 0.5602 (0.5514-0.5689) | 10.10 | 0.66/0.43 | 0.09 | < 0.001 |
| BMI | 0.5612 (0.5524-0.5700) | 24.78 | 0.60//0.49 | 0.09 | < 0.001 |
| TyG-BMI | 0.6047 (0.5962-0.6131) | 1042.03 | 0.59/0.56 | 0.15 | Reference |
| **MI** |  |  |  |  |  |
| TyG | 0.5882 (0.5664-0.6101) | 10.16 | 0.69/0.50 | 0.19 | < 0.001 |
| BMI | 0.5839 (0.5617-0.6060) | 26.33 | 0.46/0.67 | 0.13 | < 0.001 |
| TyG-BMI | 0.6066 (0.5849-0.6283) | 1042.63 | 0.60/0.55 | 0.15 | Reference |
| **Stroke** |  |  |  |  |  |
| TyG | 0.5536 (0.5432-0.5639) | 10.10 | 0.66/0.43 | 0.09 | < 0.001 |
| BMI | 0.5512 (0.5408-0.5616) | 24.22 | 0.67/0.41 | 0.08 | < 0.001 |
| TyG-BMI | 0.5919 (0.5819-0.6019) | 1042.15 | 0.59/0.56 | 0.15 | Reference |
| **HF** |  |  |  |  |  |
| TyG | 0.5540 (0.5368-0.5712) | 10.40 | 0.46/0.62 | 0.08 | < 0.001 |
| BMI | 0.5690 (0.5513-0.5866) | 24.77 | 0.62/0.49 | 0.11 | < 0.001 |
| TyG-BMI | 0.6345 (0.6182-0.6508) | 1060.05 | 0.62/0.58 | 0.20 | Reference |

Caption: CVD, cardiovascular disease; MI, myocardial infarction; HF, heart failure; TyG, triglyceride-glucose index; BMI, body mass index; Cum TyG-BMI, cumulative triglyceride-glucose-body mass index.

**Table S3. Sensitivity analysis excluding outcome events occurring within the first year of follow-up (n=227)**

|  | **Q1** | **Q2** | **Q3** | **Q4** |
| --- | --- | --- | --- | --- |
| Case/Total | 466/11,877 | 762/11,839 | 911/11,836 | 1,148/11,798 |
| IR | 4.00 | 6.73 | 8.20 | 10.72 |
|  | 1.00 | 1.29 (1.15, 1.45) | 1.31 (1.17, 1.47) | 1.43 (1.27, 1.60) |

The model was adjusted for age, sex, HDL-C, LDL-C, hs-CRP, eGFR, current smoker current drinker, physical activity, hypertension, diabetes mellitus, hypoglycemic drugs, anti-hypertensive drugs, lipid-lowering drugs, and the baseline TyG-BMI value in 2010.

Caption: IR, Incidence rate, per 1000 person-years.

**Table S4. Sensitivity analysis excluding participants using antihypertensive, antidiabetic, and lipid-lowering medications**

|  | **Q1** | **Q2** | **Q3** | **Q4** |
| --- | --- | --- | --- | --- |
| **Participants who used antihypertensive drugs were excluded (n=6,341)** |  |  |  |  |
| Case/Total | 412/11,214 | 649/10,711 | 724/10,177 | 845/9,134 |
| IR | 3.74 | 6.33 | 7.55 | 10.14 |
|  | 1.00 | 1.32 (1.17, 1.50) | 1.33 (1.18, 1.51) | 1.52 (1.34, 1.72) |
| **Participants who used Hypoglycemic drugs were excluded (n=1,807)** |  |  |  |  |
| Case/Total | 466/11,756 | 770/11,607 | 890/11,401 | 1,094/11,006 |
| IR | 4.05 | 6.96 | 8.33 | 10.98 |
|  | 1.00 | 1.33 (1.18, 1.49) | 1.34 (1.19, 1.50) | 1.48 (1.32, 1.67) |
| **Participants who used Lipid-lowering drugs were excluded (n=406)** |  |  |  |  |
| Case/Total | 477/11,851 | 806/11,803 | 964/11,795 | 1,226/11,722 |
| IR | 4.11 | 7.17 | 8.75 | 11.60 |
|  | 1.00 | 1.34 (1.19, 1.50) | 1.35 (1.21, 1.51) | 1.48 (1.32, 1.66) |

The model was adjusted for age, sex, HDL-C, LDL-C, hs-CRP, eGFR, current smoker, current drinker, physical activity, hypertension, diabetes mellitus, hypoglycemic drugs, anti-hypertensive drugs, lipid-lowering drugs, and the baseline TyG-BMI value in 2010.

Caption: IR, Incidence rate, per 1000 person-years.
